# Roles for Selenoprotein I and Ethanolamine Phospholipid Synthesis in T Cell Activation

**DOI:** 10.3390/ijms222011174

**Published:** 2021-10-16

**Authors:** Chi Ma, Verena Martinez-Rodriguez, Peter R. Hoffmann

**Affiliations:** Department of Cell and Molecular Biology, John A. Burns School of Medicine, University of Hawaii, Honolulu, HI 96813, USA; chima@hawaii.edu (C.M.); verenam@hawaii.edu (V.M.-R.)

**Keywords:** selenium, selenoprotein, seleocysteine, autoimmunity, lymphocyte

## Abstract

The selenoprotein family includes 25 members, many of which are antioxidant or redox regulating enzymes. A unique member of this family is Selenoprotein I (SELENOI), which does not catalyze redox reactions, but instead is an ethanolamine phosphotransferase (Ept). In fact, the characteristic selenocysteine residue that defines selenoproteins lies far outside of the catalytic domain of SELENOI. Furthermore, data using recombinant SELENOI lacking the selenocysteine residue have suggested that the selenocysteine amino acid is not directly involved in the Ept reaction. SELENOI is involved in two different pathways for the synthesis of phosphatidylethanolamine (PE) and plasmenyl PE, which are constituents of cellular membranes. Ethanolamine phospholipid synthesis has emerged as an important process for metabolic reprogramming that occurs in pluripotent stem cells and proliferating tumor cells, and this review discusses roles for upregulation of SELENOI during T cell activation, proliferation, and differentiation. SELENOI deficiency lowers but does not completely diminish de novo synthesis of PE and plasmenyl PE during T cell activation. Interestingly, metabolic reprogramming in activated SELENOI deficient T cells is impaired and this reduces proliferative capacity while favoring tolerogenic to pathogenic phenotypes that arise from differentiation. The implications of these findings are discussed related to vaccine responses, autoimmunity, and cell-based therapeutic approaches.

## 1. Introduction

Selenium (Se) is an essential dietary trace mineral that is important for various aspects of human health, including optimal immunity [1]. The biological effects of Se are mainly exerted through its incorporation into selenoproteins as the 21st amino acid, selenocysteine (Sec) [2]. The 25 members of the selenoprotein family exhibit a wide variety of functions including the control of reactive oxygen species and cellular redox tone, regulating thyroid hormone metabolism, facilitating sperm maturation and protection, and promoting optimal immunity [3]. Under conditions of low Se status, the translation of selenoproteins stalls at the Sec-encoding UGA codon and both the mRNA and truncated protein become degraded through nonsense-mediated decay and destruction via C-end degrons, respectively [4,5]. In a Se deficient individual, the brain, muscle, and testes receive ‘priority’ for bioavailable Se at a cost to other tissues, such as those comprising the immune system [6]. Insufficient Se intake or other factors (e.g., defects in selenoprotein gene expression or some chronic infections that deplete Se) can impair adaptive immunity, especially T cell responses that are critical for producing effective vaccine responses and fighting infections [7,8]. Interestingly, not all types of immune responses are equivalently affected by Se deficiency or by Se supplementation [9,10]. Although the reasons for this are unclear due to an inadequate understanding of the mechanisms by which Se affects the immune system, data have recently emerged regarding roles for individual selenoproteins in immunity. This is particularly the case for T cell immunity, and a better understanding of how selenoproteins regulate T cell immunity may provide new targets for therapeutic intervention for immune based disease.

## 2. T Cell Immunity and Selenoprotein I Expression

CD4^+^ T cells constitute the topmost regulatory layer of the adaptive immune system, providing cytokine ‘help’ to CD8^+^ T cells (effector cells of cell mediated immunity) and B cells (effector cells of humoral immunity), thus coordinating acquired immune responses [11]. CD4^+^ T cells are activated through the T cell receptor (TCR), proliferate and differentiate into helper subsets, which forms the foundation for their ability to shape immune response and mediate host protection [12]. CD8^+^ T cells are also activated through their TCR to produce effector and memory cells required for optimal immunity [13]. Levels of Se and selenoproteins regulate T cell functions that drive both cell-mediated and humoral immunity [14,15,16,17]. However, it remains unclear which selenoproteins are involved in the different steps of T cell activation, proliferation, and differentiation. To gain insight into the roles that different selenoproteins play in T cell activation, we used real-time PCR to evaluate the selenoprotein transcriptome in naïve vs. activated T cells purified from C57BL/6 mice [18]. Results showed that a subset of selenoprotein mRNAs increased during T cell activation. These included the thioredoxin reductases (TXNRD1-3) enzymatically regenerate reduced thioredoxin and promote reducing capacity within cells, and we previously published that mRNA levels for these enzymes were increased as a mechanism for regulating redox tone in during T cell activation [7]. An interesting result found during T cell activation was an increase in the mRNA encoding selenoprotein I (SELENOI), which was upregulated nearly 3-fold [18]. Protein levels and enzyme activity for SELENOI were similarly increased in activated T cells. This raised the question: Why do SELENOI levels increase during T cell activation and what is its role in regulating T cell immunity?

To understand how SELENOI is involved in T cell activation, a bit more background on this selenoprotein is necessary. After the initial identification of the SELENOI gene in 2003 [19], the sequence was subsequently characterized through a homology search that found the cytidine diphosphate (CDP) alcohol phosphatidyltransferase signature, a common motif conserved in phospholipid synthases [20]. This study went on to demonstrate that SELENOI exhibits ethanolamine phosphotransferase (Ept) activity in vitro, providing the first data showing that SELENOI (which this group called EPT1) is an enzyme that transfers phosphoethanolamine from CDP-ethanolamine to 1,2-DAG acceptors to produce phosphatidylethanolamine (PE). SELENOI is only found in vertebrates, and in humans is expressed in a wide variety of cells [19,20]. A more recent study showed that fibroblasts from a patient with a mutation (exon skipping) leading to nonfunctional SELENOI had impaired Ept activity and reduced levels of several PE species, especially plasmenyl PE [21]. This study focused on the neurodevelopment defects exhibited by the patient, and another earlier clinical report substantiated the effects of a Arg112Pro mutation in the gene encoding SELENOI on the central nervous system (CNS) [22]. Understandably, these patients with severe CNS impairments were not evaluated in terms of immune cell function. Furthermore, there has been a paucity of data published regarding dietary Se regulating less overt changes in SELENOI. This is significant because the central nervous system retains Se under conditions Se deficiency, at a cost to the immune system [6]. Thus, individuals deficient in Se may maintain higher SELENOI levels in the brain compared to the immune system, and Se deficient T cells expressing lower levels of SELENOI may impact immunity. SELENOI protein or enzyme activity have not been measured in individuals with different Se status, but these data would be useful in determining how Se intake is related to function for this selenoprotein.

## 3. Structure of SELENOI Related to the Synthesis of PE and Plasmenyl PE

**Figure 1:**
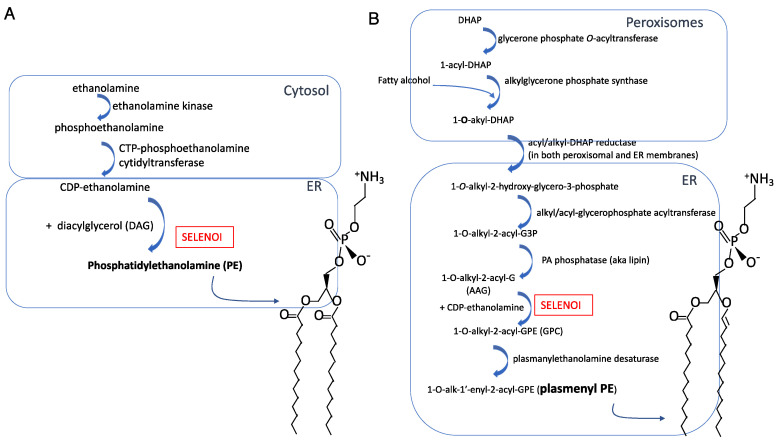
Synthesis pathways and molecular structures for (**A**) PE and (**B**) plasmenyl PE.

Plasma membrane phospholipids are distributed asymmetrically between the outer and inner leaflets of viable mammalian cells. The outer leaflet of the plasma membrane is composed primarily of sphingomyelin (SM) and phosphatidylcholine (PC), whereas the inner leaflet contains phosphatidylserine (PS) and phosphatidylethanolamine (PE) and phosphatidylinositol (PI), with cholesterol distributed equally [23,24]. In the plasma membrane and organelle membranes of mammalian cells, PE comprises 15–25% of total phospholipids [25]. PE is comprised of 1,2-diacylglycerol (DAG) and ethanolamine phosphate (Figure 1A). A structurally related ethanolamine phospholipid, plasmenyl PE, is an ether-linked lipid comprised of 1-alkyl-2-acylglycerol (AAG) and ethanolamine phosphate (Figure 1B), and is found at a lower abundance in cellular membranes compared to PE. SELENOI is involved in de novo synthesis of both PE and plasmenyl PE, carrying out the transfer of phosphoethanolamine from cytidine diphosphate (CDP)-ethanolamine to DAG to generate PE or to AAG to generate plasmenyl PE. In particular, SELENOI catalyzes the final step of the Kennedy pathway in the interface of the cytosol and endoplasmic reticulum (ER) that is responsible for synthesizing PE [26]. Although an alternative pathway operates in the mitochondria for synthesizing PE from PS, the importance of SELENOI in the synthesis of PE for cellular membranes through the Kennedy pathway has been clearly demonstrated in primary human fibroblasts and HeLa cells [21]. Plasmenyl PE is synthesized through a separate pathway beginning in peroxisomes (Reactions 1–3) and finishing in the ER membrane (Reactions 4–7). SELENOI catalyzes the sixth reaction to generate plasmenyl PE [27]. The vinyl ether bond at the sn-1 position of glycerol backbone contributes to a difference in biophysical properties compared to PE, and plasmenyl PE species are enriched in lipid rafts [28,29,30]. Thus, synthesis of PE and plasmenyl PE species are largely dependent on SELENOI and serve structural functions in cellular membranes, but possible roles in signaling pathways and in metabolic regulation are beginning to emerge [31,32]. Related to SELENOI (aka ethanolamine phosphotransferase 1 or EPT1) is the enzyme choline/ethanolamine phosphotransferase (CEPT1). CEPT1 is not a selenoprotein and can use both CDP-ethanolamine and CDP-choline as substrates, while SELENOI only uses CDP-ethanolamine [6]. This seems to suggest that SELENOI uses the selenocysteine residue to select cytidine diphosphate (CDP)-ethanolamine instead of CDP-choline as a substrate, although there are no data to include or exclude this possibility. Experimental evidence strongly suggests that CEPT1 can partially compensate for a lack of SELENOI expression [33], and PE may be generated by converting other phospholipids like phosphatidylserine to PE [34]. This other pathway occurs within the mitochondrial inner membrane, involving the conversion of the serine base to ethanolamine by PS decarboxylase (PSD) [26]. The PS decarboxylation pathway generates different PE species from CDP-ethanolamine pathway (i.e., the Kennedy pathway) [35]. This implies that phospholipid synthesis may occur by different routes and highlights the priority that cells place on maintaining balanced membrane composition.

**Figure 2:**
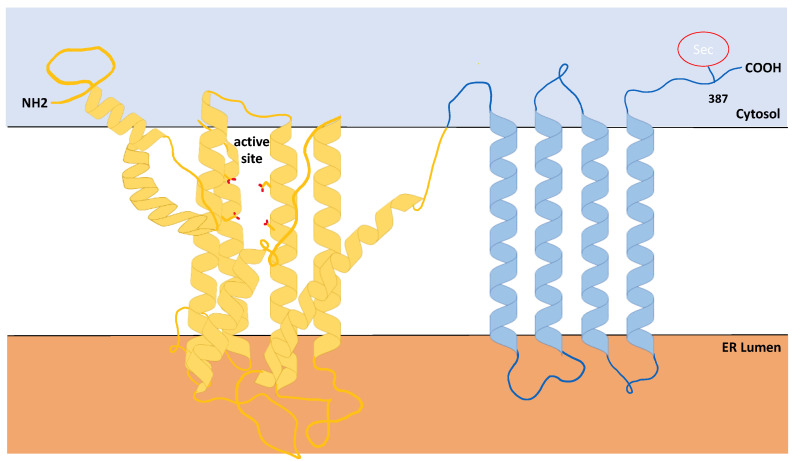
Predicted structure of Selenoprotein I. Results from Phyre Alarm and other online prediction programs show that, similar to other phosphotransferases, SELENOI is predominantly comprised of hydrophobic amino acids (~90%) with the catalytic domain residing within a membrane pocket. Note the C-terminal Sec residue is located outside of the catalytic domain.

As discussed above, SELENOI belongs to the family of selenoproteins and also to the family of phospholipid transferases. The latter enzymes are identified by a conserved CDP-alcohol phosphotransferase motif D(X)_2_DG(X)_2_AR(X)_7-12_G(X)_3_D(X)_3_D (X represents any amino acid and subscript numbers represent number of residues) commonly shared by all enzymes catalyzing the biosynthesis of phospholipids [20,36]. SELENOI is a predicted transmembrane protein that is fully embedded into the lipid bilayer, with its transmembrane helices traversing the membrane multiple times, as shown in Figure 2 [37]. Since most phospholipid transferases reside primarily in the (ER) membranes [38], SELENOI was initially thought to share the same localization. Recent studies showed that SELENOI is mainly localized in the Golgi apparatus [33], although these data involved overexpressed, tagged SELENOI in a cell line. Similar to other phospholipid transferases, the catalytic domain of the enzyme is predicted to be located within a pocket of the lipid bilayer that is accessible from the cytoplasm [37]. In redox selenoenzymes, selenocysteine residue is located within the catalytic domain in a prototypical C-X-X-U motif (X represents any amino acid), and replacement of selenocysteine with cysteine has been shown to reduce the catalytic activity of some of these enzymes [39,40]. In contrast, SELENOI does not contain a C-X-X-U motif and SELENOI’s selenocysteine is located near the C-terminus at amino acid position 387 that is predicted to reside in the cytosol, apart from active site in within the membrane pocket [37]. This raises the question if selenocysteine is necessary for the catalytic function of this enzyme? Overexpression of SELENOI cDNA in a bacterial expression system led to a truncated form of the protein due to the lack of recognition of eukaryotic SECIS elements, and this Sec-deficient protein retained in vitro Ept activity [20]. This suggests the selenocysteine residue is not directly involved in the catalytic function of SELENOI, which is further supported by the observation described above that CEPT1 (that lacks a selenocysteine residue) may exert Ept activity when compensating for a lack of SELENOI.

## 4. Cellular Membranes and SELENOI KO

**Figure 3:**
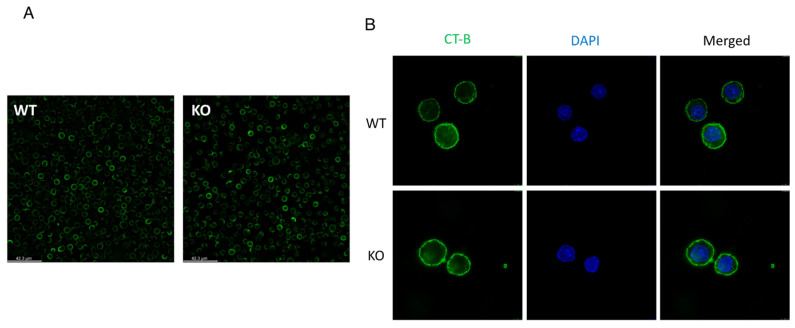
Lipid raft staining is similar between SELENOI KO T cells and WT controls. Mouse T cells isolated from mice and activated through the TCR for 24 h were stained for lipid rafts using standard fluorescent cholera toxin subunit B protocols. (**A**) Fluorescent microscopy images from live cells at 20× and (**B**) confocal microscopy images of paraformaldehyde fixed cells at 63× reveal similar patterns of staining between KO and WT T cells.

Published data show that SELENOI KO does not affect TCR induced signaling [18], which is somewhat surprising given that SELENOI deficiency decreases phospholipids involved in cellular membrane structure. In particular, the ratio of PE levels relative to PC levels plays a key role in membrane fluidity or rigidity [28,41]. Sufficient membrane rigidity is crucial in TCR signaling strength required to drive proliferation and differentiation [42]. Thus, one may expect a disruption of TCR signaling or, at a minimum, weaker TCR signaling with the lowered PE and plasmenyl PE levels in T cells that accompanies SELENOI KO. The lack of an effect of SELENOI KO on TCR signaling may suggest a compensatory process serves to maintain cellular membrane integrity in the absence of SELENOI. There is also a question regarding lipid rafts, which are fluctuating nanoscale assemblies of sphingolipid, cholesterol, and proteins that can be stabilized to coalesce, forming platforms that function in membrane signaling and trafficking [43]. Lipid rafts are enriched for plasmenyl PE [44], so it would reason that SELENOI deficiency in T cells that reduces plasmenyl PE should affect lipid raft organization. However, membrane raft organization does not appear differ between SELENOI KO versus WT control T cells as determined by fluorescent staining (Figure 3). This is consistent with the lack of effect of SELENOI KO on TCR signaling described above. Overall, there is insufficient evidence to date suggesting that membrane integrity or structure is affected by SELENOI KO in T cells.

## 5. Metabolic Reprogramming During T Cell Activation Requires SELENOI for Proliferation

In the non-activated or quiescent state, T cells mainly exhibit catabolic activity involving the break-down of nutrients to fuel cell survival. Upon T cell receptor (TCR) triggered activation, T cells transition to a state of anabolism in which nutrients are used to construct the molecular building blocks that are incorporated into cellular biomass to support proliferation [45,46]. In addition to small molecule precursors, energy is also needed for proliferation. This requirement is met during TCR-induced activation through increased glucose uptake via upregulated glucose transporters, which is accompanied by induced aerobic metabolism [47]. These changes are akin to shifts in cancer cell metabolism known as the Warburg effect. TCR-induced metabolic reprogramming is critical for generating sufficient energy and precursor molecules for subsequent rounds of mitosis, which promotes the T cell expansion phase that is so crucial for immune clearance of pathogens. The balance of catabolic and anabolic pathways in a cell determines how much adenosine triphosphate (ATP) is generated versus consumed, the availability of biosynthetic precursors, and the redox status of the cell [48]. Redox status may be controlled by antioxidant and redox regulating selenoproteins, with free thiols playing a key role [16]. However, SELENOI is an unconventional selenoprotein that is not involved in redox reactions. Since SELENOI is directly involved in two different anabolic pathways, one for PE synthesis and the other for plasmenyl PE synthesis, it follows that this selenoprotein is likely an integral part of the metabolic reprogramming during T cell activation.

To understand the role of SELENOI in T cell proliferation, our research group conducted loss-of-function studies in T cells isolated from different transgenic mouse models. In particular, an inducible knockdown (KD) mouse model along with a T cell specific knockout (KO) mouse model were compared to wild-type (WT) controls for TCR-induced proliferative capacity. SELENOI KD led to a ~22% decrease and KO to a ~56% decrease in proliferation compared to WT controls [18]. In vivo T cell expansion to vaccination was also decreased in T cell specific SELENOI KO mice compared to WT controls. Surprisingly, the TCR signaling was not affected by SELENOI deficiency and levels of PE and plasmenyl PE were only partially decreased. The latter observation may be explained by the fact that enzyme activity of a related phospholipid transferase may compensate for PE and plasmenyl PE when SELENOI is absent [33]. The most impressive result of SELENOI deficiency in activated T cells was a progressive accumulation of ATP, which was detected by the metabolic sensor AMPK and led to lower activation of this kinase. In fact, treating WT T cells with the AMPK inhibitor, dorsomorphin, reduced proliferation by similar levels as SELENOI KO. These data collectively suggest that SELENOI serves a critical function during T cell activation to maintain ethanolamine phospholipid synthesis and thereby keep a balanced metabolism within the cells as they undergo metabolic reprogramming. Removing SELENOI causes a ripple effect-these synthesis pathways are disrupted and this causes ATP to accumulate, which is sensed within the cells by AMPK and eventually proliferation is reduced.

## 6. SELENOI and T Helper Cell Differentiation

As proliferation takes place, the fates of the daughter T cells differ through asymmetric cell division and differentiation [49]. In particular, naïve CD4^+^ T cells differentiate into one of several T helper cell lineages depending on signals from antigen presenting cells and cytokines present in the surrounding environment. In addition to these external stimuli, internal factors, such as cellular metabolism, can influence differentiation [50]. CD4^+^ T cell subsets express unique sets of cell surface markers and transcription factors, while secreting a defined array of cytokines that determines their functional properties [51]. It was recently demonstrated that CD4^+^ T cell differentiation was dependent on ethanolamine kinase 1, CTP-phosphoethanolamine cytidyltransferase, and SELENOI enzymes that comprise the CDP-ethanolamine pathway for de novo synthesis of PE and plasmenyl PE [52]. In particular, this pathway was important for T follicular helper (T_FH_) cell differentiation by promoting the surface expression and functional effects of the chemokine receptor, CXCR5. Unlike our SELENOI KO T cells that were decreased, but not lacking, PE and plasmenyl PE as described above, these studies largely focused on T cells lacking these phospholipids. A state of complete loss of PE and plasmenyl PE in T cells is unlikely given the essential roles these phospholipids play in development [53,54], but the importance of ethanolamine phospholipid synthesis in T_FH_ cell formation does highlight how certain metabolic pathways promote T cell differentiation outcomes over others.

T helper type 17 (Th17) cells represent another subtype of T cells differentiating from naïve Th cells that promote inflammation. Th17 cells are required for immune responses to specific extracellular bacteria and fungi [55], but dysregulated Th17 responses may also contribute to pathogenesis in autoimmune diseases, such as rheumatoid arthritis and multiple sclerosis (MS) [56,57]. In contrast, CD4^+^ regulatory T (Treg) cells contribute to the suppression of immune responses and immune homeostasis, countering Th17 cells to protect against autoimmune disorders. Treg differentiation relies on the upregulation of the transcription factor FoxP3, and Treg cells function to secrete immunosuppressive cytokines TGF-β and IL-10 [58]. A major factor influencing Th17/Treg fates during T cell activation is the type of metabolic reprogramming that occurs after TCR engagement, which serves to meet increased demand for energy and metabolites [59].

Th17 cells take on a distinct metabolic signature that promotes differentiation into this subtype, heavily relying on finely tuned glycolysis, glutamine metabolism, and fatty acid synthesis for their differentiation and pro-inflammatory function [60,61,62]. Sustained mitochondrial oxidative phosphorylation has also been shown to regulate the fate decision between pathogenic Th17 and Treg cells [63]. These coordinated shifts in specific metabolic pathways that occur during T helper cell differentiation may be disrupted in a number of ways, and we recently identified SELENOI as a metabolic enzyme involved in regulating T helper cell differentiation. SELENOI mRNA and protein levels increased at early stages of Th17 differentiation, similar to levels of the master regulator that drives Th17 phenotypes, RORγt (manuscript submitted). Using T cell specific SELENOI KO mice, our studies have found that naïve CD4^+^ T cells were directed toward Treg and away from Th1 and Th17 subtypes when activated through the TCR. Moreover, T cell specific KO of SELENOI protected mice from a mouse model of MS, experimental autoimmune encephalitis (EAE), showing that SELENOI deficiency reduces Th17 pathology. These data may suggest that targeting SELENOI in T cells may represent a potential therapeutic approach to treating MS. Indeed, cell-based therapies have been proposed for restoring homeostasis in MS patients such as tolerogenic dendritic cells, Tregs, mesenchymal stem cells, and vaccination with T cells [64]. Interfering with SELENOI activity to skew Th17/Treg cells toward a tolerogenic phenotype certainly presents its challenges, but may provide a new cell-based therapeutic approach for this or other autoimmune disorders.

## 7. Conclusions

**Figure 4:**
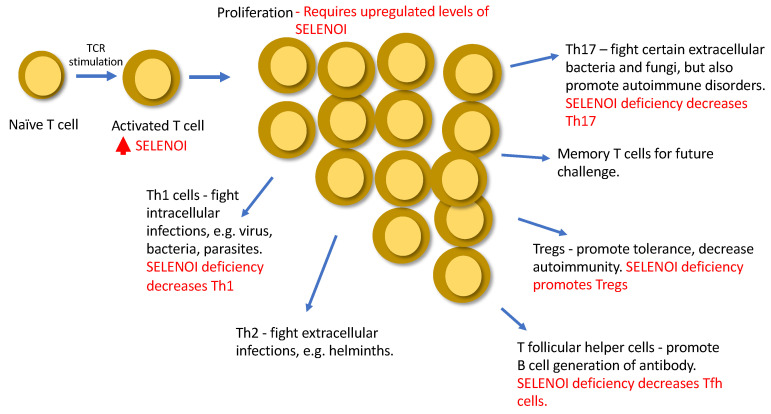
A summary of roles of SELENOI in T cells.

Overall, insights into roles for SELENOI in T cell functions have been made using SELENOI loss-of-function models (Table 1). Upregulated SELENOI triggered by TCR engagement contributes to both proliferation and differentiation, which may be crucial for a variety of immune responses (Figure 4). The emerging field of metabolic reprogramming during T cell activation is providing new insights into factors and pathways that regulate optimal responses to pathogens and tumors. SELENOI has recently been identified as an anabolic enzyme involved in metabolic reprogramming, and disruption of its activity in mouse models has been shown to effectively decrease EAE. Ethanolamine phospholipid synthesis is particularly important for metabolic reprogramming in pluripotent stem cells and proliferating tumor cells [31,65], and our recent work showed a crucial role for ethanolamine phospholipid synthesis during T cell activation. How this may be translated into new therapies remains uncertain, but targeting metabolism may be incorporated into cell-based therapies involving T cells. For example, chimeric antigen receptor (CAR) expressing T cells have become an effective approach for treating some cancers, and advances have been made to improve the metabolic fitness and efficacy of CAR T cells [66]. This may provide a framework for developing new strategies in treating diseases, including autoimmune disorders, focused on regulating T cell metabolism and providing optimal immunity.

## Figures and Tables

**Table 1:** A summary of roles for SELENOI in T cell functions.

T Cell Function	Role for SELENOI	References
TCR signaling	SELENOI KO has minimal effect on signaling pathways downstream of TCR engagement	[18]
Membrane raft distribution	SELENOI KO has minimal effect on raft distribution or membrane fluidity	Data presented herein
Metabolic reprogramming	SELENOI KO disrupts ATP generation/consumption during TCR activation	[18]
Metabolic sensing	SELENOI KO impairs sensing by AMPK during TCR activation	[18]
T cell proliferation	SELENOI KO decreases in vivo and ex vivo TCR induced proliferation	[18]
T cell differentiation	T helper cell differentiation is affected by SELENOI KO; Tfh and Th17 cells are reduced, while Treg cells are increased	[52]

## Data Availability

Not applicable.
